# Supplementary material for: A Biosensor Platform for Metal Detection Based on Enhanced Green Fluorescent Protein
Source: Sensors (Basel). 2019 Apr 18;19(8):1846. doi: 10.3390/s19081846 (PMC6514868; doi:10.3390/s19081846)
Supplement: Supplementary file 1 [file sensors-19-01846-s001.pdf]

Supplementary Information

A Biosensor Platform for Metal Detection Based on Enhanced Green Fluorescent Protein

Woonwoo Lee [1], Hyojin Kim [1], Yerin Kang [1], Youngshim Lee [2] and Youngdae Yoon [1,*]

[1] Department of Environmental Health Science, Konkuk University, 120 Neungdong-ro, Gwangjin-gu, Seoul 05029, Korea; lunia2005@hanmail.net (W.L.); gywls6772@naver.com (H.K.); yelin0514@naver.com (Y.K.); librashim@gmail.com (Y.L.); yyoon21@gmail.com (Y.Y.)

[2] Division of Bioscience and Biotechnology, Bio/Molecular Informatics Center, Konkuk University, 120 Neungdong-ro, Gwangjin-gu, Seoul 05029, Korea

* Correspondence: yyoon21@gmail.com; Tel.: +82-2-450-0443

Received: 18 March 2019; Accepted: 16 April 2019; Published: 18 April 2019

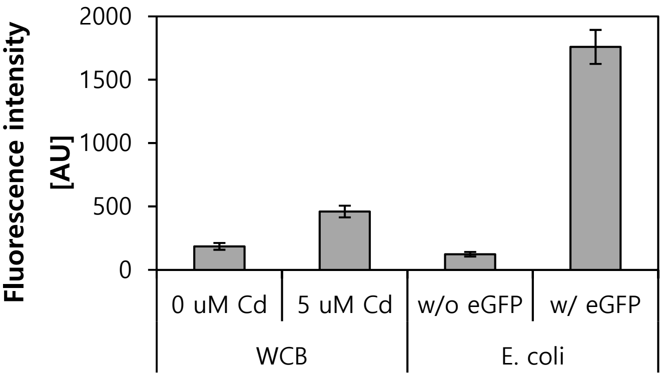

Figure S1. Original fluorescence signals from WCB with eGFP-loop 2 and wild type *E. coli* with and without eGFP in the presence of cadmium ions. eGFP-loop 2 showed signal with cadmium ion, while basal level signal that was similar level to *E. coli* without eGFP was determined without cadmium ion. *E. coli* cells with eGFP as reporter showed much strong signals.
